# Association of neutrophil to lymphocyte ratio with preterm necrotizing enterocolitis: a retrospective case-control study

**DOI:** 10.1186/s12876-022-02329-3

**Published:** 2022-05-17

**Authors:** Yuju Mu, Hua Wang

**Affiliations:** 1 Department of Pediatrics, West China Second University Hospital, Sichuan University, No. 20, section 3, Renmin South Road, Chengdu, 610041 Sichuan China; 2 Key Laboratory of Birth Defects and Related Disease of Women and Children (Sichuan University), Ministry of Education, Sichuan University, No. 20, section 3, Renmin South Road, Chengdu, 610041 Sichuan China

**Keywords:** Diagnostic tools, Necrotizing enterocolitis, Inflammation, Neutrophil to lymphocyte ratio, Predictive cutoff value, Preterm neonates

## Abstract

**Background:**

There have been few studies on the relationship between the neutrophil to lymphocyte ratio (NLR) and necrotizing enterocolitis (NEC). We conducted a retrospective case-control study to investigate this relationship in preterm neonates.

**Methods:**

A total of 199 preterm neonates diagnosed with NEC between January 2018 and January 2020 were included in this study. For each preterm infant with NEC that was admitted to the neonatal intensive care unit (NICU), controls were preterm neonates (matched for gestation and year of birth) who were not diagnosed with NEC. Exclusion criteria were post-maturity, small or large for gestational age (week of pregnancy), congenital major anomalies, and cyanotic congenital heart disease. Univariate and multivariate logistic regression analyses were used to identify the association between NLR and preterm NEC.

**Results:**

This study included 93 preterm neonates with NEC and 106 matched controls. There were no significant differences in gestational age (GA), birth weight (BW), age, sex, vaginal delivery (VD), chorioamnionitis (CA), and gestational diabetes mellitus (GDM) between the groups. Compared with the control group, the lower and higher NLR levels in the NEC group were statistically different. Following univariate analysis, NLR was a risk factor for NEC (odds ratio [OR], 1.40; 95% confidence interval [CI], 1.00–1.90; *P* = 0.042), and according to multivariate analysis, risk factors for NEC were NLR ≥ 3.20 and NLR < 1.60, within 1 week before NEC diagnosis. Thus, NLR values of ≥ 1.60 and < 3.20 were determined as the predictive cut-off values for protecting preterm infants from NEC (Model I: OR, 0.20; 95% CI, 0.10–0.40; *P* < 0.001) and (Model II: OR, 0.10; 95% CI, 0.00–0.40; *P* < 0.001].

**Conclusions:**

NLR ≥ 1.60 and NLR < 3.20 were associated with a decreased risk of NEC in preterm infants.

## Background

Necrotizing enterocolitis (NEC) is the most common and potentially devastating multifactorial life-threatening condition affecting the gastrointestinal tract (GIT) in premature infants; it is a leading cause of morbidity and mortality in infants born between 23 and 28 weeks of gestation. It has been reported that NEC prevalence is 5–7% in very low birth weight (VLBW) infants [1], 20–30% of whom eventually die [2]. Moreover, NEC survivors have a high risk of severe long-term complications, such as short bowel syndrome, growth delay, and neurodevelopmental sequelae, which are associated with a low quality of future individual life and increasing long-term social health care costs [3]. It is a multifactorial disease in which the exact pathogenesis remains elusive. Hypoperfusion of the intestines, immaturity of the intestinal barrier system, selection and colonization of harmful bacteria in the gut, bacterial translocation, infection, and inflammatory response all contribute to the disease.

The Bell’s staging criteria for NEC were first devised in 1978 and modified by Walsh and Kliegman in 1986 [4]; almost all neonatologists and medical journals use the modified version. Mild or suspected NEC (Bell’s Stage I) comprises mild systemic symptoms and mild nonspecific intestinal symptoms [5–7]. Since these nonspecific symptoms are often observed in infants with extremely low birth weights in the neonatal intensive care unit (NICU), it is very difficult to distinguish between NEC and feeding intolerance, as well as other gastrointestinal diseases and sepsis. Moderate or definitive NEC (Bell’s Stage II) further includes radiological findings and moderate systemic signs [5–7]. Nonetheless, due to the inter-observer variability, radiological findings cannot accurately predict NEC diagnosis [8]. While the early clinical signs of NEC are usually very discrete and nonspecific, the use of Bell’s criteria based solely on clinical and radiographic features has significant limitations [9]. Considering these limitations for early NEC diagnosis, research has focused on the discovery of biomarkers capable of prediction, early diagnosis, and discrimination of NEC from other intestinal diseases.

The severity of NEC differs, ranging from mild involvement that could be managed solely by fasting for bowel rest or using antibiotics, to severe intestinal necrosis requiring surgical treatment. Since intestinal inflammation of NEC cannot generally be controlled by either conservative or surgical treatment satisfactorily, efforts should be focused on the importance of preventing NEC and improving NEC diagnostic capabilities. Finding a diagnostic method with high specificity and sensitivity to identify preterm NEC earlier is an immediate priority.

Therefore, we tried to find specific biomarkers associated with these conditions to improve the NEC diagnostic capabilities. In this context, a biomarker could be defined as any measurable parameter that provides meaningful information regarding the diagnosis of NEC [10]. During the neonatal period, noninvasive and easy-to-use biomarkers that can accurately determine NEC are limited. In the present clinical practice, biomarkers currently include acute phase proteins, inflammatory mediators, and immunoreactive molecules. Nonetheless, the early prediction and diagnosis of NEC, or the ability to discriminate different stages of NEC correctly from other gastrointestinal diseases, remains unresolved [3]. To our knowledge, there was no single biomarker or cluster of biomarkers that meet the neonatologists’ satisfaction.

In some recent studies, researchers focused on noninvasive approaches [11]. Complete blood count-derived parameters and their relation to certain diseases have recently received attention from researchers [12]. Neutrophils are an essential factor in the innate immune response during inflammation. Increased neutrophil levels elicit an appropriate inflammatory response in patients with mild-to-moderately severe disease. One study investigated the incidence of neutropenia (neutrophil counts of ≤ 1000 cells/mL) in small for gestational age (SGA) neonates and found that newborn infants have a four-fold increased risk of developing NEC [13]. Regardless, neutrophils may express excessive inflammatory cytokines that contribute to excessive inflammation and tissue damage. Lymphocytes are also involved in the immune response against bacterial and viral infections [14]. During an inflammatory response, the number of neutrophils increases or decreases, while lymphocytes decrease in number. The neutrophil to lymphocyte ratio (NLR) reflects changes in neutrophil and lymphocyte levels, indicating the presence of inflammation. It has been shown that NLR is superior to white blood cell (WBC) in the prediction of adverse outcomes in a variety of inflammatory and surgical conditions. By using NLR, it is possible to have an idea about two different immune pathways; the first one is neutrophil which is accountable for continuing inflammation and the second one is lymphocytes that shows regulatory pathways [15]. Therefore, the combination of neutrophil and lymphocyte concentrations indicated as NLR may be more valuable as a marker of inflammation than neutrophilia, neutropenia, or lymphocytopenia alone for predicting bacterial infections. Studies suggest NLR is associated with occult inflammation in certain conditions, and it has also been shown to be useful in predicting adverse outcomes in patients with pancreatitis, appendicitisand other critical conditions [16]. The clinical use of NLR has been shown in bacterial pneumonia, and it was reported that NLR was significantly.

elevated in COVID-19 patients [17]. Moreover, the NLR has been used as a reliable marker for inflammation and as a prognostic index for a variety of medical conditions, including ischemic stroke, cerebral hemorrhage, major adverse cardiac events, and solid tumors [18]. A study found that the maternal NLR can independently predict the risk of NEC in very preterm infants [19].

Since there is a strong association between inflammation and NEC, and between inflammation and NLR, we aimed to investigate the relationship between NLR and NEC in preterm neonates. We hypothesized that NLR was associated with an increased risk of NEC based on analyses of existing clinical data.

## Methods

### Study population

**Fig. 1:**
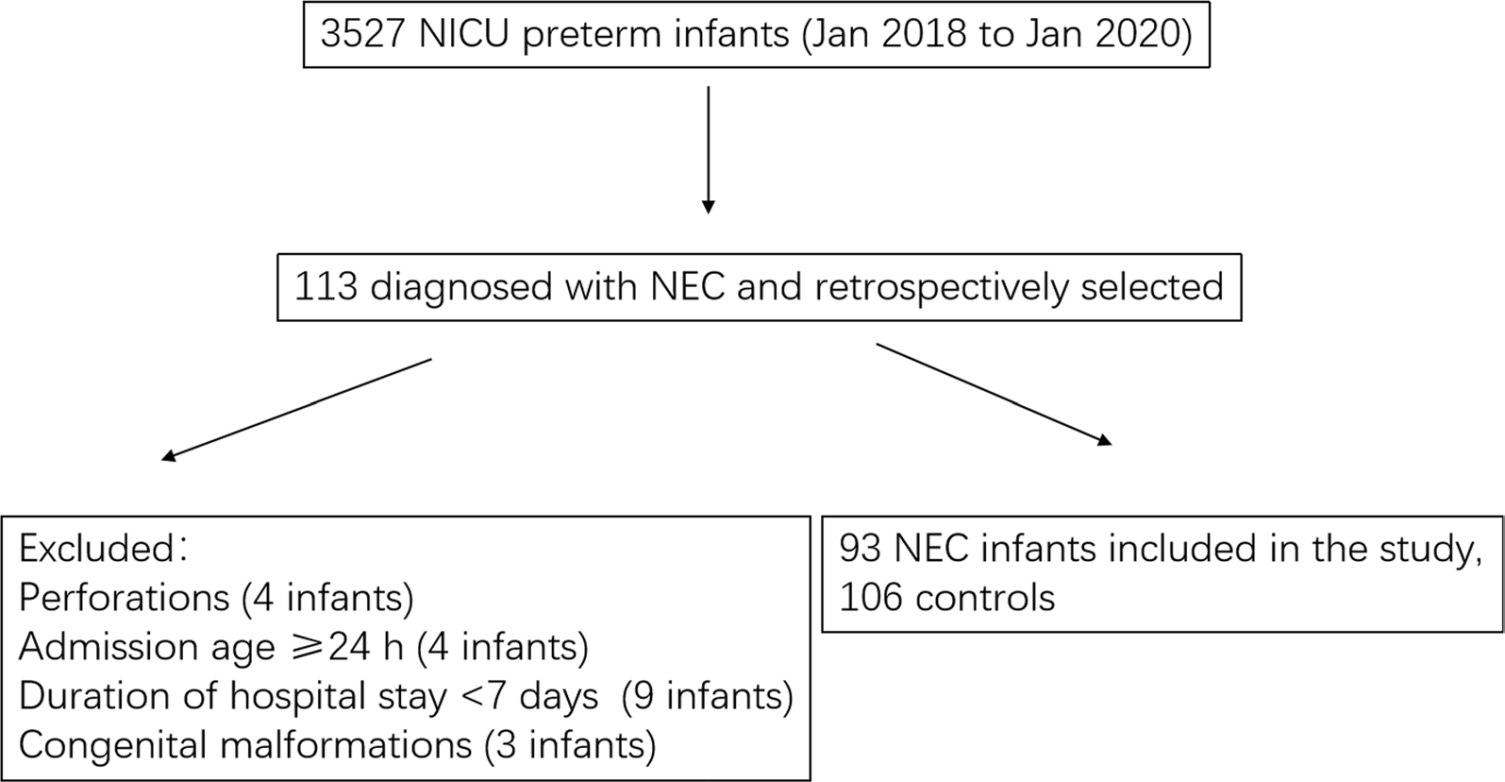
Patients selection flow chart (2018–2020)

The study population consisted of preterm infants who developed NEC over 2 years from January 2018 to January 2020 at the NICU of the West China Second University Hospital, Sichuan University. Preterm infants diagnosed with NEC who showed perforations (4 infants), had an admission age of ≥ 24 h (4 infants), had a hospital stay of < 7 days (9 infants), and those with congenital malformations (3 infants) were excluded. The case group included 93 preterm infants who met the criteria for NEC; the control group included 106 preterm infants matched for gestational age and year of birth (Fig. 1).

### Study design

We performed a retrospective case-control study. For this analysis, NEC diagnoses were made based on the presence of clinical, radiological, and/or histopathological evidence that fulfilled the Bell’s modified criteria. The clinical data, including data pertaining to the demographical characteristics and comorbidities of the mother, was collected from the patients’ electronic medical records.The NLR was determined using the mean neutrophil and lymphocyte counts from all blood tests within 1 week before the patient’s NEC diagnosis. Initial laboratory investigations included the WBC count, platelet (PLT) count, and C-reactive protein (CRP) levels.

### Statistical analysis

For descriptive analyses, categorical variables and continuous variables were described as percentages and mean (standard deviation), respectively. The distribution of each covariate of the exposed and non-exposed groups was compared using the t-test (normal distribution) or the Kruskal–Wallis rank sum test (non-normal distribution) for continuous variables and the chi-square test for categorical data (Table 1). Next, univariate logistic regression (Table 2) and multivariate logistic regression models (Table 3) were used to examine whether the NLR was associated with NEC in preterm neonates. Statistical results were displayed as an odds ratio (OR), with their corresponding 95% confidence interval (CI). All analyses were performed using R (http://www.R-project.org) and Empowerstats software (www.empowerstats.com, X & Y Solutions, Inc.)

**Table 1:** Baseline characteristics of the study participants (N = 199)

NEC	Case group (n = 93)	Control group (n = 106)	Standardize diff	*P*-value
Gestational age, weeks (Mean ± SD)	32.74 ± 2.52	32.46 ± 2.75	0.10 (− 0.17–0.38)	0.466
Birth weight, g (Mean ± SD)	1733.83 ± 504.75	1793.36 ± 581.09	0.11 (− 0.17–0.39)	0.444
Age, hours (Median[Q1-Q3])	0.42 (0.35–0.87)	0.48 (0.36–0.94)	0.00 (− 0.28–0.28)	0.993
SEX (n, %)			0.18 (− 0.10–0.46)	0.209
Male	43 (46.24%)	59 (55.14%)		
Female	50 (53.76%)	47 (44.86%)		
NLR (Mean ± SD)	2.10 ± 1.40	1.70 ± 0.50	0.40(0.10–0.70)	0.008
CRP, mg/L, (Median[Q1-Q3])	4.89 (1.26–18.00)	3.30 (2.33–6.13)	0.48 (0.20–0.76)	< 0.001
VD (n, %)			0.08 (− 0.20–0.36)	0.577
No	71 (76.34%)	78 (72.90%)		
Yes	22 (23.66%)	28 (27.10%)		
CA (n, %)			0.02 (− 0.26–0.29)	0.907
No	54 (58.06%)	63 (58.88%)		
Yes	39 (41.94%)	43 (41.12%)		
PE (n, %)			0.38 (0.10–0.66)	0.008
No	40 (43.01%)	66 (61.68%)		
Yes	53 (56.99%)	40 (38.32%)		
GDM (n, %)			0.27 (− 0.01–0.55)	0.061
No	46 (49.46%)	67 (62.62%)		
Yes	47 (50.54%)	39 (37.38%)		

For explanation of abbreviations, see the main text

**Table 2:** Univariate analysis for necrotizing enterocolitis

	Statistics	OR (95% CI)	*P-*value
NLR	1.90 ± 1.10	1.40 (1.00–1.90)	0.042
NLR tripartite group			
Low	66 (33.16%)	1.00	
Middle	66 (33.16%)	0.00 (0.00–0.10)	< 0.001
High	67 (33.68%)	0.90 (0.40–2.00)	0.756
NLR threshold value group			
< 1.60	97 (48.70%)	1.00	
≥ 1.60, < 3.20	71 (35.70%)	1.60 (0.52–4.93)	0.413
≥ 3.20	31 (15.60%)	9.00 (1.65–49.14)	0.011
Gestational age, weeks (Mean ± SD)	32.59 ± 2.64	1.04 (0.94–1.16)	0.464
Birth weight, g (Mean ± SD)	1765.68 ± 546.38	1.00 (1.00–1.00)	0.442
CRP (n, %)	8.86 ± 13.72	1.05 (1.02–1.08)	0.002
CA (n, %)			
No	117 (58.79%)	1.00	
Yes	82 (41.21%)	1.03 (0.59–1.82)	0.907
PE (n, %)			
No	106 (53.27%)	1.00	
Yes	93 (46.73%)	2.13 (1.21–3.76)	0.009
GDM (n, %)			
No	113 (56.78%)	1.00	
Yes	86 (43.21%)	1.71 (0.97–3.01)	0.062

For explanation of abbreviations, see the main text

**Table 3:** Relationship between the neutrophil to lymphocyte ratio and necrotizing enterocolitis according to different models

Variable	Crude Model^a^		Model I^b^		Model II^c^	
	OR (95% CI)	*P*-value	OR (95% CI)	*P*-value	OR (95% CI)	*P*-value
NLR	1.40 (1.10–1.90)	0.010	1.70 (1.20–2.40)	0.003	1.60 (1.10–2.40)	0.013
NLR value group						
< 1.60	1.00		1.00		1.00	
≥ 1.60, < 3.20	0.30 (0.10–0.50)	< 0.001	0.20 (0.10–0.40),	< 0.001	0.10 (0.00–0.40)	< 0.001
≥ 3.20	1.00		1.00		1.00	

For explanation of abbreviations, see the main text

^a^Crude Model not adjusted

^b^Model I adjusted for GA, BW, VD

^c^Model II adjusted for GA, BW, sex, VD, CA, PE, GDM, and CRP

## Results

### Baseline characteristics

This study was conducted with 199 preterm neonates that were appropriate for gestational age (AGA). Of these, 93 were diagnosed with preterm NEC, and 106 were preterm infants matched for gestational age and year of birth. The general characteristics of the study population are summarized in Table 1. Overall, infants who were diagnosed with NEC had a higher NLR value (2.10 ± 1.40 vs. 1.70 ± 0.50; *p* = 0.008) and CRP value (4.89 [1.26–18.00] vs. 3.30 [2.33–6.13] mg/L; *p* < 0.001). In addition, preeclampsia (PE) was more common in the NEC group (53/93 vs. 40/106; *p* = 0.008). Apart from these three factors, there was no noticeable difference between the two groups regarding gestational age (GA) (32.74 ± 2.52 vs. 32.46 ± 2.75 weeks; *p* = 0.466), birth weight (BW) (1733.83 ± 504.75 vs. 1793.36 ± 581.09 g; *p* = 0.444), age (0.42 [0.35–0.87] vs. 0.48 [0.36–0.94] hours; *p* = 0.993], sex (male: female, 43:50 vs. 59:47; *p* = 0.209), VD (22/93 vs. 28/106; *p* = 0.577), CA (39/93 vs. 43/106; *p* = 0.907), and gestational diabetes mellitus (GDM) (47/93 vs. 39/106; *p* = 0.061).

### Association of NLR levels with NEC

To investigate the association of NLR levels with NEC, subjects were divided into three groups according to NLR tertiles. Univariate analysis showed that the NLR was significantly correlated with preterm NEC (odds ratio [OR], 1.40; 95% confidence interval [CI], 1.00–1.90; *P* = 0.042). In addition, the CRP value (OR, 1.05; 95% CI, 1.02–1.08; *P* = 0.002) and PE (OR, 2.13; 95% CI, 1.21–3.76; *P* = 0.009) might also be associated with preterm NEC (Table 2).

**Fig. 2:**
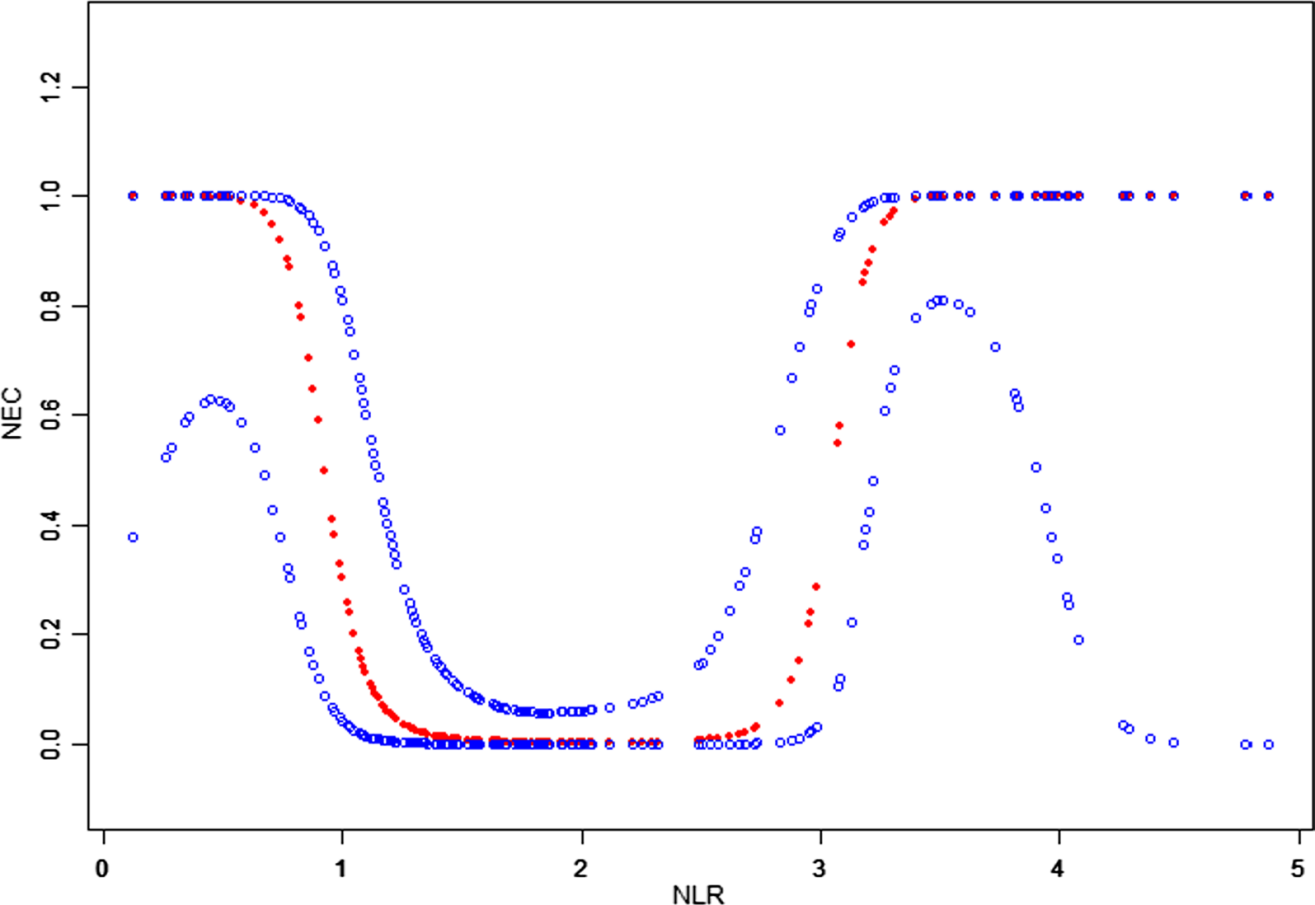
Association between neutrophil to lymphocyte ratio and necrotizing enterocolitis. A threshold, nonlinear association between neutrophil to lymphocyte ratio (NLR) and necrotizing enterocolitis (NEC) was determined (*P* < 0.05) from a generalized additive model (GAM). The red line represents the smooth curve fit between variables. The blue bands represent the 95% confidence interval from the fit

After multivariable risk adjustment for potential confounding factors (Table 3), including GA, BW, sex, and VD in Model I and GA, BW, sex, VD, CA, PE, GDM, and CRP in Model II, the NLR was still positively associated with NEC in preterm neonates. In addition, an NLR of ≥ 1.60 and an NLR of < 3.20 within 1 week before NEC diagnosis could significantly decrease the risk of preterm NEC (Model I: OR, 0.20; 95% CI, 0.10–0.40, *P* < 0.001) and (Model II: OR, 0.10; 95% CI, 0.00–0.40; *P* < 0.001). Therefore, NLR values of ≥ 1.60 and < 3.20 were determined as the predictive cutoff values for the preterm NEC group. A threshold, nonlinear association between NLR and NEC was observed in a generalized additive model (GAM) (Fig. 2).

## Discussion

The incidence of NEC is extremely high in preterm infants [20], and is associated with an increase in mortality. Most of the survivors often experience a variety of serious short and long-term complications, such as intestinal stenosis, short bowel syndrome, and neurological sequelae [21, 22]. Although the literature is limited, the direct hospital cost of NEC has been estimated to be appropximatety between 1.4 to over 10 times higher in VLBW infants with NEC than in VLBW infants without NEC. The increased cost stems from longer hospital stays and additional medical interventions (e.g., surgery, central line placement, and increased total parenteral nutrition time), as well as the increased risk of morbidities associated with NEC. Therefore, NEC has a significant negative impact on healthcare utilization and costs. Therefore, a method to reduce the NEC occurence rate will not only prevent the associated mortality and morbidity and improve the neonatal prognosis, but also reduce healthcare and social costs.

The uncertainty in the course of NEC is due to the absence of a definitive etiology and pathogenesis; moreover, it manifests in a variety of ways. The signs and symptoms of NEC may be concealed and nonspecific, making it difficult to diagnose preterm neonates with NEC, earlier. Due to a deficiency in available diagnostic skills and tools, and the accelerated progression of the disease, some infants, particularly those who are premature, do not receive timely treatment. In practice, the diagnosis and treatment of NEC involve duplicate blood testing and abdominal X-rays, the use of broad-spectrum antibiotics, and fasting or decreased enteral feeding. Consequently, many infants may develop secondary anemia, further disturbing the gut microbiome and resulting in retarded growth and development. Therefore, it is crucial to develop strategies to identify infants who are less susceptible to NEC to avoid excessive treatment. Furthermore, to decrease the healthcare utilization and costs associated with NEC, identifying preterm infants with NEC accurately and rapidly is extremely important. For the sake of reducing the burden of NEC in preterm neonates, the prediction and early diagnosis of this catastrophic disease are of utmost necessity.

The current clinical practice to diagnose NEC depends on nonspecific systemic symptoms including inflammation, local abdominal signs, and specific radiographs to determine the presence of gastrointestinal inflammation. It is important to note that all of these symptoms are nonspecific for NEC, thus, confusing it with the differential diagnosis of other conditions, such as neonatal sepsis, other gastrointestinal diseases, and feeding intolerance. When NEC is suspected, the modified Bell’s staging criteria should be applied, which allows rapid clinical decision-making. The features of the Bell’s staging criteria represent clinical, laboratory, and radiologic signs, most of which are nonspecific and may be less sensitive [23], and there are numerous shortcomings in the current use of Bell’s staging criteria [6, 20]. The criteria should not be used as a prognosticating diagnostic tool, but only if NEC has already occurred. The ideal diagnostic biomarker should be both highly sensitive, so as not to miss potential cases, and specific to avoid over-treating infants who are not likely to progress to NEC. Moreover, it should be reliable and have accurate predictive value. Other useful features include affordability, reproducibility, and availability [24]. Some researchers have investigated biomarkers as possible tools to predict NEC, such as interleukin-6 [25], intestinal fatty acid-binding protein [26], and serum amyloid A [27]. Regardless, the majority of these are not available for routine laboratory tests performed at most medical institutions because of medical costs and the complex methodology required. On the contrary, complete blood counts are simple, easy, and convenient to determine. So blood NLR is a simple sign of clinical inflammation.

The increase in a patient’s neutrophil count and decrease in lymphocyte count is a response to microbial infection. The increase in the number of neutrophils is due to a reduction in neutrophil apoptosis and rapid mobilization of neutrophils from a marginated pool within the bone marrow [28–30]. Neutrophils are important in removing pathogens, but neutrophil infiltration and activation also result in major tissue injury associated with acute and chronic inflammatory disorders [31]. Although neutrophils play a vital role in host defense, they can also cause severe morbidity and mortality. The lymphocyte count decreases due to the migration of activated lymphocytes to inflamed tissues and increased apoptosis of lymphocytes [29, 32]. It indicates immunosuppression and plays a role in the septic patients’ mortality [33, 34]. Zahorec previously introduced the NLR as a simple, rapid, and cost-effective method to determine inflammation in critically ill patients [35]. In addition, a previous study showed that this ratio could be utilized as a predictor of disease severity in adult patients [36]. Recent studies found that NLR had a higher sensitivity and specificity for diagnosing infectious diseases [37, 38]. For example, Sen et al. showed that using the NLR preoperatively could be a promising predictor of bacteremia and postoperative sepsis in patients requiring percutaneous nephrolithotomy [39]. In China, studies have highlighted similar results, where Yang et al. found that the NLR was significantly higher in the death group than in the control group within 205 adult bloodstream infection patients [40]. In summary, NLR could be utilized to indicate the status of the inflammatory response and the level of physical stress in a timely and accurate manner [40]. In addition, NLR could be used as a predictive marker for patients with infections.

NEC is a disease induced by multiple factors, we only hope that we can find some indicators which could avoid overtreatment for preterm NEC to help clinicians make the right decision. Thus, the influence of some confounding factors, such as gestational age, nutrition and nursing factors were excluded by statistical analysis using R (http://www.R-project.org). In our study, a statistically significant positive correlation was found between the NLR and preterm NEC when NLR values were ≥ 1.60 and < 3.20. In the univariate analysis, NLR was significantly correlated with preterm NEC (OR, 1.40; 95% CI, 1.00–1.90; *P* = 0.042). Moreover, CRP and PE may also be associated with preterm NEC. After adjusting for these potential confounders in the multivariate logistic regression analysis, we still found a significant correlation between NLR and preterm NEC. NLR values of ≥ 1.60 and < 3.20 were determined as the predictive cutoff values for the preterm NEC group (OR, 0.20; 95% CI, 0.10–0.40; *P* < 0.001), which is associated with a decreased risk of NEC in preterm infants. In addition, NLR (< 1.60 or ≥ 3.20) may be used as a diagnostic tool for preterm NEC. NLR is is a rapid, inexpensive, and useful indicator that could be estimated via the complete blood count. Cost-effective and easy to-assess nature of this test may contribute to its utility in clinical practice. This ratio may be applied in clinical practice and can be used during routine diagnostic processes for preterm NEC in NICUs.

This study has some limitations. The inherent bias due to the retrospective design of the study should be mentioned. Moreover, the sample number of this study was small and only from a single center, which may also restrict the accuracy and generalizability of the results.

## Conclusions

In conclusion, the NLR, an easy, simple, inexpensive, and rapid tool, could be used, in advance, to predict preterm NEC along with other biomarkers. Timely NEC prediction would not only allow for high-risk neonates to receive preventive treatments such as early exposure to the mother’s colostrum, careful nutritional consideration, use of probiotics, and increased skin-to-skin care [41], but also decrease unnecessary antibiotic therapy or even surgical interventions.

Overall, our findings emphasize the necessity to improve medical measures to decrease the incidence of preterm neonates NEC. Future prospective studies with a larger population of preterm infants are required to validate the results of this study. In addition, in the forthcoming studies, we plan to develop a predictive model of the NLR for NEC diagnosis through machine learning, and then prospectively substantiate the model’s accuracy using a larger number of preterm infants.

## Data Availability

The datasets used and/or analyzed during the current study are available from the corresponding author on reasonable request.

## Abbreviations

AGA: Appropriate for gestational age
BW: Birth weight
CA: Chorioamnionitis
CI: Confidence interval
CRP: C-reactive protein
GA: Gestational age
GIT: Gastrointestinal tract
GDM: Gestational diabetes mellitus
GAM: Generalized additive model
NLR: Neutrophil to lymphocyte ratio
NEC: Necrotizing enterocolitis
NICU: Neonatal intensive care unit
OR: Odds ratio
PLT: Platelet
PE: Preeclampsia
SGA: Small for gestational age
VD: Vaginal delivery
VLBW: Very low birth weight
WBC: White blood cell

## Declarations

### Methods

All methods were performed in accordance with the relevant guidelines and regulations.
